# Disclosures of conflicts of interest from the editors of the Journal of Global Health – 2024

**DOI:** 10.7189/jogh.14.01001

**Published:** 2024-12-02

**Authors:** Harry Campbell, Igor Rudan

We continue the practice of transparently disclosing any potential competing interests for us as co-Editors-in-Chief of the Journal of Global Health, following the International Committee of Medical Journal Editors (ICMJE) Recommendations for the Conduct, Reporting, Editing, and Publication of Scholarly Work in Medical Journals [1]. As we expect our authors and reviewers to declare their possible conflicts of interest concerning the manuscript under consideration for publication in the Journal, we also annually declare our commitments and relations that may influence the editorial review process.

Except for editorials, which we consider as welcome and important contributions from the editors of any scholarly journal, we believe that any active researchers should try to avoid, whenever possible, publishing their own research articles in the journal that they (co-)edit. This is also true for us as co-Editors-in-Chief of the Journal of Global Health. Therefore, when one of the decision-making co-Editors-in-Chief submits a paper to the Journal of Global Health, it is managed and reviewed independently from this editor, who has no access to or influence on the review process and editorial decision-making. This is always indicated in the Disclosure of Interest section at the end of article, if it is eventually accepted and published.

We also wish to disclose some specific circumstances in the case of the Journal of Global Health. Since its inception, the journal has served as a ‘hub’ and a connecting point to the three international collaborations of scientists working in the field of global health: the Global Health Epidemiology Research Group (GHERG); the Child Health and Nutrition Research Initiative (CHNRI) with its priority-setting method; and the International Society of Global Health (ISoGH). This is why the Journal of Global Health typically considers papers from these large international collaborations, although the Journal’s co-Editors-in-Chief are usually among their co-authors. Those papers are treated as described above – entirely independently from the co-Editors-in-Chief. They represent a negligible proportion of papers among both the Journal of Global Health’s overall annual output and the co-Editor-in-Chiefs’ overall scientific production. Moreover, they are a product of highly internationally collaborative efforts rather than individual contributions, which further minimises the risk of any conflict of interest. It is also possible that the co-Editors-in-Chief may choose to publish other types of papers in the Journal, depending on specific circumstances surrounding each such paper. These undergo the very same process listed above for the GHERG’s, CHNRI’s, and ISoGH papers, where an external editor handles the peer review process and decides independently from the co-Editor-in-Chiefs.

We declare our other conflicts of interest below, and will regularly publish updated declarations when any changes to our relationships or activities occur.

## PROFESSOR HARRY CAMPBELL, MD, FRCPE, FFPH, FRSE, FMedSci

### Personal and organisational

Professor Campbell is employed by the University of Edinburgh, where he holds a position as Professor in the Usher Institute, College of Medicine and Veterinary Medicine. He is also co-Director of the National Institute for Health and Care Research (NIHR) Global Respiratory Health Unit. He currently receives research funding from the World Health Organization (WHO), the UK NIHR, and the Baszucki Brain Research Fund. He has received consultancy payments, paid via the University of Edinburgh, from the WHO, the Bill and Melinda Gates Foundation, the UK Funding Councils (Research Excellence Framework), and Sanofi in the past 10 years.

### Journal

Professor Campbell holds a position as the co-Editor in Chief. He occasionally reimburses expenses related to Journal’s work, including travel and consumables, and is a member of the ISoGH.

### Unpaid positions

Numerous technical advisor appointments with the WHO in the past 10 years.

## PROFESSOR IGOR RUDAN, MD, MSc, DSc, PHD, MPH, FRSE, MAE, MEASA

### Personal and organisational

Professor Rudan is employed by the University of Edinburgh, where he holds a position as Professor and Co-Director of the Centre for Global Health Research. He is also co-Director of the WHO Collaborating Centre in Population Health Research and Training. He is currently spending his sabbatical leave at the Oxford University (1 October 2024 to 15 September 2025).

### Journal

Professor Rudan holds a position as the co-Editor in Chief. He occasionally reimburses expenses related to Journal’s work, including travel and consumables, and serves as the President of the ISoGH.

### Paid positions

Professor Rudan has occasional technical advisor appointments to the WHO, the United Nations Children’s Fund (UNICEF), and the World Bank.
